# Supplementary material for: Revisiting the ownership effect in adults with and without autism
Source: PLoS One. 2023 Dec 22;18(12):e0293898. doi: 10.1371/journal.pone.0293898 (PMC10745184; doi:10.1371/journal.pone.0293898)
Supplement: Attachment — Submitted filename: response to reviewers-points.docx [file pone.0293898.s001.docx]

Dear Dr Johannes Hönekopp

Please find attached a copy of the revised manuscript, “**Revisiting the ownership effect in adults with and without autism**”. This is a revision of manuscript number PONE-D-23-08426, as invited by yourself.

We are very grateful to you and the two expert reviewers of the previous submission for your helpful comments. These have given us excellent guidance on how to  improve the paper, and we feel that it is much improved because of it. Below we provide a detailed summary of how we have addressed the individual comments raised by each reviewer and yourself. We have also revised the formatting of the manuscript and referencing style throughout, in line with PLOS ONE style requirements. All changed text is highlighted in yellow in the revised manuscript.

Reviewer #1:
Page 5, paragraph 2: Typo: “Firstly, in neurotypical adult studies have found…” – omit “in”?

We have corrected this typo.

The introduction provides detailed discussion of studies investigating the ownership effect in memory tasks. This is understandable given the authors’ task. However, the ownership effect has been investigated in other ways that would be worth highlighting – particularly as the strength of the effect may differ across methodologies. For example, in Hartley and Fisher (2018), autistic children clearly did not display a mere ownership effect when valuing/trading toys they were actually given to own and keep. It would be interesting to consider here – and perhaps revisit in the General Discussion in light of the results – how/why the ownership effect might differ across samples of autistic children/adults when tested on a memory task versus object preference/valuation tasks

Thank you for highlighting this. We have extended our discussion of the boundary conditions and potential mechanisms of the ownership effect in the Introduction and Discussion.

There is a recent and relevant study that the authors may (or may not) wish to mention in their introduction: Hartley & Leeming (2022, Cog Dev) Could sensitivity to object authenticity be developmentally delayed in autism? Here, autistic adults were asked to value a range of hypothetical objects, including items belonging to the self. For the most part, neurotypical and autistic adults did not differ in how they valued items associated with self. The groups differed more in terms of how they valued objects belonging to other people.

Thank you for suggesting this research; we have added this citation to the discussion.

In Experiment 1, the sample’s mean AQ score is 16.98. It would be helpful to indicate how this compares with the sample in Grisdale et al. (2014) that comparisons are being drawn with. Did the samples differ in their distribution of AQ scores and could this potentially explain differences in findings?

Thank you for raising this. We have added a footnote to Methods section, noting that, “Grisdale et al. (2014) included 40 (2 male; 38 female) neurotypical undergraduate students aged 18-24 years, with a mean AQ score of 12.52 (SD = 5.94; range: 2-23). Thus, our mean AQ score is higher and the range is larger than that in Grisdale et al (indicating greater autistic traits and greater variance in our sample)”.

I was somewhat surprised to see that only 52% of participants showed an ownership effect. How does this compare with rates in other studies employing similar methodologies? Notably, this proportion seems much lower than what we see in other tasks assessing ownership effects (e.g. studies involving ownership of real objects).

We have added a note regarding the difference in rates between studies: “…the ownership effect size observed in the current study (*η_p_^2^* = 0.06; 52% of participants displayed an ownership effect) is smaller than that observed by Grisdale et al. (2014) (*η_p_^2^* = 0.72; 95% of participants displayed an ownership effect)”.

In this Discussion, or perhaps the General Discussion, I think it would be worth reflecting on this point and considering to what extent this kind of memory task actually taps into psychological effects associated with ownership.

We have added a paragraph at the end of the general Discussion to relate the findings to psychological mechanisms.

The ownership effect categorical classification percentages for the neurotypical (71.43%) and autistic participants (65.29%) in Experiment 2 are quite a bit higher than that of the much larger sample of neurotypical participants in Experiment 1 (52%). Why might this be? The comparisons between correct hit rates across samples and conditions are interesting. In Experiment 1, the difference between corrected hit rates for self and other items in a sample of 100 neurotypical adults was 0.022 (0.547 vs. 0.525). In Experiment 2, the difference between corrected hit rates for self and other items in a sample of autistic adults was 0.015 (0.38 vs. 0.365), just 0.007 less. Thus, the effect of Referent seems pretty similar between these two groups across Experiments. The difference between corrected hit rates for self and other items in the neurotypical sample in Experiment 2 was 0.066 (0.551 vs 0.485), which is larger than the difference for the autistic group but also larger than the difference for the neurotypical group in Experiment 1. These comparisons between groups require unpacking. Why is the Referent effect larger for neurotypical participants in Experiment 2 relative to Experiment 1, and why is it similar for autistic and neurotypical participants across studies?

Thank you for raising this. We now highlight this point in the Discussion for Experiment 2, and note that differences in sample characteristics (including student sample in Experiment 1 and a higher overall group mean AQ) is likely to explain differences in classification %.

It strikes me that the biggest, and perhaps most meaningful, difference between autistic and neurotypical groups is in overall corrected hit accuracy – the corrected hit rates for both self and other items in the autistic sample are substantially lower than those observed in the two neurotypical samples. This point is not discussed at present, but warrants detailed theoretical consideration.

We have extended the discussion for Experiment 2 to highlight that when the main effect of group was broken down to look at the group x referent interaction, there is no significant between group difference in memory accuracy for other owned items, but there is for self-owned items. This suggests that the impairment is in self-processing but not other processing.

Page 22, paragraph 2: I couldn’t really understand the authors’ point “Second, experimental designs are developed to, and hence become popular if, they produce robust/replicable effects – i.e., effects that are low in between-subject variance. However, low variance between-subjects makes these tasks unreliable/less effective for detecting individual differences (i.e. a correlational approach) which are dependent on this between-subject variance (Hedge et al., 2018). Therefore, it is also possible that some or all the tasks in this thesis were unsuitable for an individual differences approach.” The memory task used in these experiments surely generated sufficiently high variance between participants, making it ideal for examining individual differences? The reported data certainly don’t seem to be characterised by floor or ceiling effects.

Thank you for pointing this out. We agree that this point was unclear and have removed it from the manuscript.

Building on paragraph 2 on page 23, it would be beneficial to reflect on how and why ownership effects may differ across tasks and the implications for studying autistic and neurotypical children/adults. How do the memory findings reported in this study fit in with the broader literature examining ownership effects in autism? Overall, how might the influence of ownership on cognition differ for autistic people?

We have added this additional reflection on effects in autism to the General Discussion, and added discussion linking these effects to language and ToM in autism.

Page 24, paragraph 2: The theoretical explanation in this paragraph requires more detail. Why might differences in the ownership effect in autism be explained by differences in understanding ownership rights? The ownership effect measured in this study is a memory effect, whereas awareness of ownership rights is underpinned by sensitivity to cultural rules. Also, how might impairments in Theory of Mind increase the difficulty of mentally representing and tracking relationships between people and objects? I can see how differences in mentalistic reasoning might impact on understanding of feelings/emotions towards objects held by the self or others, but couldn’t tracking of people-object relationships be subserved by basic statistical learning which is typically intact in autism? That being said, a recent study by Hartley and Leeming (2022, JADD) suggests that autistic children may have difficulty tracking these invisible relationships when objects look similar, and don’t identify their objects with increased accuracy like neurotypical children do. A stronger link could also be drawn between understanding of possessive pronouns and identification of relationships between people and property (see Hartley, Harrison & Shaw, 2021, JADD). –

Thank you for pointing out this gap. We have significantly expanded the Discussion to discuss the findings in relation to theories/models of ToM and language, and have included a more direct explanation of how this might have differed between studies.

Page 24, paragraph 3: Given the similarity between corrected hit rates for neurotypical participants in Experiment 1 and autistic participants in Experiment 2, I’m not sure it is entirely accurate to say that these findings indicate that “self-referential cognition is impaired in autistic adults”. As noted above, the most prominent difference in the autistic group appears to be generally less accurate memory in the task for items associated with both the self and others.

We have replaced ‘impaired’ with “diminished” throughout the manuscript. While we agree that the main effect of group in Experiment 2, showing that corrected hit rate was greater for the neurotypical than the autistic, the key finding is that this effect was qualified by a group x referent interaction, meaning that this reduced accuracy in ASC is only significant in the self-owned trials and not in the other-owned trials (so not an overall reduction in memory accuracy in ASC).

Reviewer #2:

As a first general comment, I think there could be a somewhat more elaborate discussion of the current state of research on the self in autism (not just in relation to autistic traits), and how these results fit into that bigger picture, as the ownership paradigm is of course only one of several existing self-bias measures.

We have included additional studies using case control designs, including where no between group difference was found in self-biased perception (Williams et al. 2018), and highlight where further case-control studies are needed.

In a footnote, it is mentioned that the referent x group interaction was not present when using d’. I think discussing this would be worthwhile, also because, given the large percentage of autistic participants showing an ownership effect, the absence of a significant difference in corrected hit rate in that group seems surprising.

Thank you for highlighting this. We agree that this non-significant main effect of group and interaction on d’ is surprising given the significant effects on these terms in the main ANOVA of corrected hit rate. This pattern suggests that the two diagnosis groups may differ in their response biases to self- and other-owned items in this task, but not in their sensitivity (i.e. the two groups are comparable in discriminating the signal for self-owned items better than for other-owned items). While we note this effect in a footnote for transparency, we focus our discussion on the results from corrected hit rates because this is the measure used in previous studies.

From the scatterplot, there seems to be one autistic individual showing a particularly strong ‘opposite’ ownership effect, could this have influenced results?

Thank you for highlighting this data point. We have re-run the analysis without this outlier and the results are the same, thus this outlier is not having a significant impact on the results and we have retained in the full sample.

In the Discussion, there is some redundant text (repetition) on page 20 (start of the first and second paragraph), I think this could be shortened.

We have revised this section to remove redundancies.

As an explanation for the discrepancy between findings of Exp 1 and Exp 2, the authors mention that the task may be less reliable/effective in detecting individual differences. However, the data show otherwise, with only 52% actually showing the effect and quite large variation. It would be good to mention this, as it makes this explanation for this specific task less likely.

We have revised this section to remove the discrepancy.

Minor comments:

Introduction, page 3: I understand the choice of ASC in the light of the terminology discussion, but when discussing the DSM diagnosis, it would be good to mention that in the DSM, the official term is ASD?

We have replaced ASC with autism spectrum disorder in the participant section when outlining the DSM diagnosis inclusion criteria.

Introduction, page 4: ‘smaller the ownership effect’: the word ‘the’ is missing.

We have corrected this typo.

For the sentence after (about autism as a spectrum and variation in the population), it would be good to add a reference.

We have added relevant references.

Methods, page 10: a sentence is presented twice here.

We have removed this repeated sentence.

Methods, page 11: What ‘stretched beta prior width’ was chosen for the Bayesian analyses for correlations?

All Bayes factors compare to the null rather than compare to the best model.

Intermediate discussion, page 13: it replicates the patterns reported in previous studies, but were effect sizes also similar?

We have clarified in the manuscript (Experiment 1) that, “It is important to note however, that the ownership effect size observed in the current study (*η_p_^2^* = 0.06; 52% of participants displayed an ownership effect) is smaller than that observed by Grisdale et al. (2014) (*η_p_^2^* = 0.72; 95% of participants displayed an ownership effect).”, and for Experiment 2 that “It is important to note that the Group x Referent effect observed in the current study (*η_p_^2^* = .08) is smaller than that observed by Grisdale et al. (2014) (*η_p_^2^* = .39).”

Discussion, page 26: Do the authors have subscale data for the AQ scores in this study? In this case, could the proposed analysis (investigating the association of subscales with the ownership effect) not be done as an exploratory analysis on these data?

We do not have subscale data for the full set of participants.
