# Supplementary material for: Revisiting the ownership effect in adults with and without autism
Source: PLoS One. 2023 Dec 22;18(12):e0293898. doi: 10.1371/journal.pone.0293898 (PMC10745184; doi:10.1371/journal.pone.0293898)
Supplement: Attachment — Submitted filename: Response to reviewers.docx [file pone.0293898.s002.docx]

Dear Dr Johannes Hönekopp

Please find attached a copy of the revised manuscript, “**Revisiting the ownership effect in adults with and without autism**”. This is a revision of manuscript number PONE-D-23-08426R1, as invited by yourself.

We are very grateful to the expert reviewer (Dr Calum Hartley) for their helpful comments. These have given us excellent guidance on how to improve the paper, and we feel that it is much improved because of it. Below we provide a detailed summary of how we have addressed the individual comments raised. All changed text is highlighted in yellow in the revised manuscript.

* Page 4 paragraph 2: When describing Grisdale et al. (2014) it is stated that neurotypical participants showed a 9% advantage in memory for self-owned items over other-owned items. When describing the results of the autistic participants, it would be helpful to also include a % for accurate comparison.

This has been added.

* Page 4 paragraph 2: New text at the end of para 2 seems to be presented in a smaller font.

This has been corrected.

* Page 5 paragraph 2: Possible typo “Firstly, neurotypical adults studies…” perhaps reword to “Firstly, studies of neurotypical adults”?

This has been corrected.

* Page 5 paragraph 2: With the added text, sentence 2 is extremely long. Recommend breaking up by starting a new sentence from “Additionally, in the perceptual domain…”

This has been corrected.

* Page 11, footnote: For readers who are unfamiliar with d-prime, it might be worth providing a brief definition and explanation for its relevance to this study.

We have added a definition to the footnote on page 10-11.

* Page 12 paragraph 1: as “data” is plural, “The data was…” should be reworded to “The data were…”. Also, the next sentence is missing a possessive apostrophe on participants (e.g. “participants’”).

This has been corrected.

* Page 13 paragraph 2: The authors’ added note about how the ownership effect rate observed in their sample differs from that reported in Grisdale et al.’s sample is helpful (although it would be worth specifying if the 95% refers to their neurotypical participants). After highlighting this discrepancy, it would be worth providing some explanation here – why was the rate observed in this study so much lower in comparison with Grisdale et al.?

We have added an explanation to highlight likely causes for between-study differences in the size of the effect on page 12: “This difference is likely explained by subtle differences between studies in task instructions, the larger sample size in our study compared to Grisdale et al. (N=100 *vs.* 40), and sample demographics.”

* Page 14 paragraph 2: The opening sentence for Experiment 2 is very long and grammatically complex. Recommend breaking up and deleting “- i.e. facilitated by alternative cognitive mechanisms –“ to improve flow.

This has been corrected.

* Page 17 paragraph 1: remove = from “p = < .001”.

This has been removed.

* Page 19 paragraph 1: I appreciate that the authors now compare categorical percentages of participant groups across experiments. However, the reasoning for similarities/differences are rather speculative and don’t seem particularly convincing. Regarding the difference between corrected hit rates for neurotypical participants in Experiments 1 and 2, it is stated that “This might be partially driven by differences in sample characteristics – in particular, the sample in Experiment 1 was made up entirely of undergraduate students, and furthermore had a higher average AQ (M = 16.98; SD = 6.51; ranging from 2-43) than the neurotypical group in Experiment 2 (M = 14.38; SD = 6.36; ranging from 3-28), both of which might have influenced differences in attention/performance on the task.” Why would we expect undergraduate students to be less likely to show a memory ownership effect than chronologically older non-students? The difference between AQ scores also seems to be an unlikely influence given the absence of statistical relationships between this measure and task performance throughout this study. Also, the autistic participants’ AQ scores in Experiment 2 were much higher and yet a larger % of this group showed an ownership effect than the neurotypical adults in Experiment 1. I recommend that the authors revisit their interpretation here and provide more detailed explanation for their reasoning.

Thank you for pointing out this inconsistency in our explanation. We have revised this paragraph as follows: “These differences might be influenced by differences in sample characteristics. In particular, the neurotypical sample in Experiment 1 was made up entirely of undergraduate students who completed the task for course credit, while participants in Experiment 2 were recruited from the Autism Research Kent participant database (made up of members of the local community who are interested in research) and were paid for their time. These different sample contexts might have influenced participants’ attention/performance on the task due to differing levels of motivation (Hess, 2014; Freund, 2008).”

* I think the authors can comfortably claim that the ownership memory effect is reduced in their autistic participants on the basis that corrected hit rate for self-owned items was significantly greater than corrected hit rate for other-owned items in neurotypical participants only. However, the theoretical explanation that this is due to specific deficits associated with self-processing in autism – argued throughout the general discussion – somewhat hinges on p = .06 indicating no population differences for other-owned items. The actual p value may even be lower if reported to 3 dp and the Bayes Factor of 1.72 falls into the category of ‘firm evidence for a difference’ (the same category as 2.31 which is interpreted as a difference in Experiment 1). The groups also did not differ in terms of the proportion of participants showing an ownership effect (the % of autistic participants showing an ownership effect was actually higher than the % of neurotypical participants in Experiment 1). Given the fragility of this evidence, claims for specific deficits in self-processing really ought to be tempered as these data may in fact be indicating that the autistic participants’ average memory accuracy was reduced across the board. I would like to see some acknowledgement and interpretation of this alternative (and arguably more likely) possibility.

Thank you for raising this important distinction. The group difference in memory for other-owned items has a p-value of 0.063 (we now report this to three decimal places in the paper for clarity). We have also expanded the discussion of Experiment 2 to highlight the subtleties in effects across different measures, and propose the possibility that a more general memory accuracy impairment might exist in the autistic group.

* page 21 paragraph 1: As the participant made physical contact with both self- and other-owned items while sorting them, it doesn’t seem relevant to mention contamination when explaining the ownership effect in the context of this study. I also question whether the extended-self hypothesis is relevant here given that participants did not actually own any of the items in this study. Is it really the case that merely sorting a card into a bucket notionally associated with the self is sufficient to transform the card into a marker of one’s personal identity?

We have removed this section from the manuscript.

* Page 24 paragraph 2: I am still questioning the theoretical interpretation presented here. Awareness of ownership rights is underpinned by sensitivity to cultural rules, so I’m not sure about its relevance. The cited evidence for differences in understanding ownership rights in autism is reported in a paper that tested children with developmental delays. As such, I’m not convinced that this evidence applies to this study’s sample of autistic adults without developmental delays. Likewise, I still have reservations about the authors’ interpretations regarding Theory of Mind (ToM). I don’t disagree with the statement that impairments in ToM might influence awareness of mental states pertaining to owned objects, but how is this relevant to the present study which doesn’t appear to tap into mentalising? Clearer explanation is required in relation to these points, or consider omitting them.

We have cut this paragraph.

* Page 25 paragraph 2: I appreciated the authors’ more detailed explanation of how they think linguistic cues may have influenced performance across groups. While I think the cited evidence is relevant and useful in terms of situating the present study in relation to wider literature exploring ownership in autism, I am not sure whether the described findings can explain the present study’s results. Again, the mentioned difficulties associated with tracking owner-object relationships using possessive pronouns were reported in autistic children with developmental delay. Is there good reason to expect that they would apply to a sample of autistic adults with high IQ and language skills commensurate with their age? If so, then this needs stronger explanation.

We have edited these paragraphs to highlight the key arguments relating to linguistic cues and remove those that do not provide a strong enough explanation.

* Page 26 paragraph 2: The authors state that “The current finding that self-referential cognition is diminished in autistic adults is a direct contrast to the patterns reported among autistic children, where either no group difference or an enhanced effect in the autistic group was found (12,13, Experiment 2). It is hard to reconcile these differences in terms of developmental differences (i.e. that psychological self-awareness is intact in childhood but becomes impaired in adulthood)…” These statements oppose the growing body of evidence for differences in ownership-related cognition in both autistic children and adults.

We have now qualified this discussion by adding: “Nevertheless, the results from the current study are in keeping with the growing body of evidence showing differences in autistic children/adults ownership-related cognition in *non-memory-related* tasks (10,46,48,49)” at the end of this paragraph – page 27.

* Page 27, paragraph 2: It is good to see that the authors have tried to situate their memory tasks in relation to broader ownership effects. I think it would be beneficial to take a further step by reflecting on how the presence and magnitude of between-population differences may (or may not) differ depending on whether stimuli associated with the self and others are truly owned (and why).

We have raised this discussion point on page 26.

Also, in light of the authors’ evaluative considerations in this paragraph, do they think it is in fact accurate to consider the memory phenomenon observed in these tasks to be a genuine “ownership effect”?

We have amended this point to clarify that it might reflect something qualitatively different than an ownership effect that is developed organically when items are *genuinely* owned by the self.

Additionally, the final sentence of this paragraph is a bit unclear (“Therefore, it is unclear whether diminished awareness of self vs. other characteristics would cause impaired ownership effect.”); is this referring to this study’s memory effects specifically, or ownership effects in general? And doesn’t this statement undermine the paper’s theoretical explanation for between-population differences? This paragraph would also benefit from some supporting citations.

This line has been deleted, and references have been added.

General comment: Throughout the manuscript, I would recommend reflecting on wording choices and how they might be perceived by neurodiverse readers, including those who are autistic. Wording like “impoverished self-concept” could potentially be considered offensive.

We have checked terminology throughout the paper to avoid potentially stigmatising language, replacing “impoverished” with “diminished”, and removed all mention of “deficit” and replaced it with “impairment” or “atypicality”.
